# Retraction Note: safety and reproductive performance in sows after vaccination: a randomized controlled trial

**DOI:** 10.1007/s11259-026-11556-y

**Published:** 2026-09-30

**Authors:** Diego Ferreira da Silva, Débora Rhayanne Matias, Francisco Rafael Martins Soto, Lucas Lacerda de Oliveira, Alessandra Marnie Martins Gomes de Castro

**Affiliations:** 1 University of São Paulo School of Nursing - USP - Graduate Program in Adult Health Nursing (PROESA), São Paulo, Brazil; 2 Stricto Sensu Graduate Program in Environmental and Experimental Pathology - UNIP, São Paulo, Brazil; 3 Federal Institute of Education, Science and Technology, São Roque Campus, São Paulo, SP Brazil

**Retraction Note: Veterinary Research Communications (2026) 50:494**

**https://doi.org/10.1007/s11259-026-11266-5**

The Authors have retracted this Article due to administrative issues concerning the authorization process for the disclosure of the data used in the study. The necessary approvals had not been finalised prior to publication.

All Authors agree with this retraction.

